# Epidural analgesia and mortality after colorectal cancer surgery: A retrospective cohort study

**DOI:** 10.1016/j.amsu.2021.102414

**Published:** 2021-05-19

**Authors:** Wiebke Falk, Anil Gupta, Maximilian Peter Forssten, Hans Hjelmqvist, Gary Alan Bass, Peter Matthiessen, Shahin Mohseni

**Affiliations:** a Department of Anesthesiology and Intensive Care, Orebro University Hospital, 701 85, Orebro, Sweden; b School of Medical Sciences, Orebro University, 702 81, Orebro, Sweden; c Department of Physiology and Pharmacology, Karolinska Institutet and Karolinska University Hospital, 171 77, Stockholm, Sweden; d Department of Orthopedic Surgery, Orebro University Hospital, 701 85, Orebro, Sweden; e Division of Traumatology, Surgical Critical Care & Emergency Surgery, Penn Medicine, Penn Presbyterian Medical Center, Philadelphia, USA; f Department of Surgery, Orebro University Hospital, 701 85, Orebro, Sweden; g Division of Trauma and Emergency Surgery, Department of Surgery, Orebro University Hospital, 701 85, Orebro, Sweden

**Keywords:** Epidural analgesia, Colorectal cancer, Open surgery, Minimally invasive surgery, Mortality

## Abstract

**Background:**

Epidural analgesia (EA) has been the standard of care after major abdominal surgery for many years. This study aimed to correlate EA with postoperative complications, short- and long-term mortality in patients with and without EA after open surgery (OS) and minimally invasive surgery (MIS) for colorectal cancer.

**Methods:**

Patient, clinical and outcome data were obtained from the Swedish Colorectal Cancer Registry and the Swedish Perioperative Registry. All adult patients diagnosed with colorectal cancer without metastases who underwent elective curative MIS or OS for colorectal cancer between January 2016 and December 2018 and who had data recorded in both registries, were included in the study. Data were analyzed for OS and MIS procedures separately. A Poisson regression model was used to investigate the association between EA and the outcomes of interest.

**Results:**

Five thousand seven hundred sixty-two patients were included in the study, 2712 in the MIS and 3050 patients in the OS group. After adjusting for patient specific and clinically relevant variables in the regression model, no statistically significant difference in risk for complications; 30-day, 90-day, and up to 3-year mortality following either MIS or OS could be detected between the EA+ and EA-cohorts.

**Conclusions:**

In this large study cohort, EA as part of the comprehensive care provided was not associated with a reduction in postoperative complications risk or improved 30-day, 90-day, or 3-year survival after MIS or OS for colorectal cancer.

## Highlights

• No reduction in postoperative complications with epidural analgesia.
• No reduction in short-term mortality with epidural analgesia after colorectal surgery.
• No reduction in long-term mortality with epidural analgesia in colorectal cancer.

## 1 Introduction

Epidural Analgesia (EA) reduces the stress response to surgical trauma [1], provides superior pain relief after major open surgical (OS) procedures compared to intravenous opioid analgesia [2], and may reduce opioid-mediated immune suppression [3,4]. Laparoscopic or robotically assisted minimally invasive surgery (MIS) are increasingly used in the resection of colorectal tumors [5]. MIS procedures are less traumatic than OS and are associated with less postoperative pain and overall physiologic stress [6]. Current postoperative pain management guidelines recommend EA as part of an Enhanced Recovery after Surgery (ERAS) care pathway for open colorectal cancer surgery, but not when a minimally invasive surgical approach is used [7]. However, little evidence exists associating EA with improved overall postoperative outcomes in patients undergoing surgery for colorectal cancer [8].

In anticipation of prospective, randomized controlled trials, we aimed to investigate an association of EA with complication rates and postoperative survival following elective colorectal cancer surgery, using data from the prospectively collected Swedish Colorectal Cancer Registry and the Swedish Perioperative Registry. Our hypothesis was that EA decreases risk of postoperative complications and improves survival in patients undergoing surgery for colorectal cancer.

## 2 Materials and methods

The cohorts were created by cross-referencing retrieved data from the Swedish Colorectal Cancer Registry (SCRCR) and the Swedish Perioperative Registry (SPOR) using patients’ unique social security numbers. The SCRCR, which has been recently validated, is a high-quality prospectively-collected nationwide registry with data completeness of over 99% [9]. SPOR started registering data in 2013 and today covers most hospitals in Sweden. According to its annual report, from 2016 until 2018, its coverage including all hospitals in Sweden that offer surgical services, has increased from 45% to 95% [10].

All patients ≥ 18 years old who underwent elective surgery, with curative intent, for colorectal cancer in Sweden between January 1, 2016, and December 31, 2018, were included in the current study. Patients from hospitals that did not contribute to the SPOR, and patients who were converted from minimally invasive to open surgery were excluded. We identified EA utilization from the SPOR database, while the SCRCR provided patient-level American Society of Anesthesiologists (ASA) classification, age, sex, tumor location (colon vs. rectum), cancer stage/TNM classification, neo-adjuvant and adjuvant therapy, type of surgery, early postoperative complications (within 30 days of operation), duration of hospital stay, and time of death. The principles of the Declaration of Helsinki and STROBE guidelines were adhered to while conducting this study (Supplementary Table) [11]. The work has been reported in line with the STROCSS criteria [12]. Ethical approval was obtained from the Swedish Ethical Review Authority (reference 2019–06434). The study was registered in the project database of Region Örebro County (ID 273334) [13].

### 2.1 Statistical analysis

Patients were categorized based on the surgical approach (MIS or OS) and whether they received epidural analgesia (EA^+^) or not (EA^−^). Patient characteristics and outcomes were compared between the cohorts, where continuous variables were reported as a mean and standard deviation or median and interquartile range, while categorical variables were presented as counts with percentages. If a continuous variable was normally distributed, the Student's t-test was employed to determine the statistically significant differences between the cohorts; otherwise, the Mann-Whitney *U* test was used. Pearson's chi-squared test and Fisher's exact test were applied for the same purpose with categorical variables.

The outcomes of interest were 30-day, 90-day, and up to 3-year postoperative mortality as well as postoperative complications. A Poisson regression model was employed to investigate the association between epidural analgesia and the previously listed outcomes. The Poisson regression analyses adjusted for age, sex, ASA classification, type of surgery, neo-adjuvant therapy, tumor location (colon vs. rectum), and cancer stage. We report the results as incidence rate ratios (IRR) with 95% confidence intervals (CI). Multiple imputation by chained equations was employed to compensate for missing data; logistic regression was used for binary variables, and a proportional odds model was used for ordinal variables. All missing data is presented in Table 1 and Table 2. Statistical significance was defined as a two-sided p-value less than 0.05. Analyses were performed using the statistical programming language R (R Foundation for Statistical Computing, Vienna, Austria) [14].

**Table 1:** Patient demographics and clinical characteristics.

	Minimally Invasive Surgery			Open Surgery		
	No Epidural Anesthesia N = 2317	Epidural Anesthesia N = 395	*P*-value	No Epidural Anesthesia N = 843	Epidural Anesthesia N = 2207	*P*-value
Age, mean (SD)	70.8 (±11.1)	70.5 (±11.2)	0.62	72.7 (±10.7)	71.2 (±11.2)	<0.001
Female, n (%)	1744 (49.5%)	197 (49.9%)	0.82	427 (50.7%)	1027 (46.5%)	0.046
**Type of epidural anesthesia, n (%)**			N/A			N/A
Thoracic	–	355 (89.9%)		–	2123 (96.2%)	
Lumbar	–	40 (10.1%)		–	84 (3.8%)	
**ASA Classification, n (%)**			0.160			0.007
1	354 (15.3%)	65 (16.5%)		63 (7.5%)	232 (10.5%)	
2	1335 (57.6%)	208 (52.7%)		415 (49.2%)	1140 (51.7%)	
3	599 (25.9%)	114 (28.9%)		327 (38.8%)	763 (34.6%)	
4	28 (1.2%)	7 (1.8%)		37 (4.4%)	69 (3.1%)	
5	1 (0.0%)	1 (0.3%)		0 (0.0%)	0 (0.0%)	
Missing	0 (0.0%)	0 (0.0%)		1 (0.1%)	3 (0.1%)	
**T stage, n (%)**			<0.001			0.360
T0	1 (0.0%)	1 (0.3%)		1 (0.1%)	2 (0.1%)	
T1	283 (12.2%)	26 (6.6%)		52 (6.2%)	148 (6.7%)	
T2	604 (26.1%)	146 (37.0%)		164 (19.5%)	378 (17.1%)	
T3	1176 (50.8%)	184 (46.6%)		470 (55.8%)	1195 (54.1%)	
T4	208 (9.0%)	32 (8.1%)		148 (17.6%)	457 (20.7%)	
TX	7 (0.3%)	3 (0.8%)		1 (0.1%)	3 (0.1%)	
Missing	38 (1.6%)	3 (0.8%)		7 (0.8%)	24 (1.1%)	
**N stage, n (%)**			0.820			0.320
N0	1477 (63.7%)	261 (66.1%)		516 (61.2%)	1283 (58.1%)	
N1	586 (25.3%)	92 (23.3%)		216 (25.6%)	637 (28.9%)	
N2	202 (8.7%)	37 (9.4%)		101 (12.0%)	252 (11.4%)	
NX	8 (0.3%)	1 (0.3%)		2 (0.2%)	5 (0.2%)	
Missing	44 (1.9%)	4 (1.0%)		8 (0.9%)	30 (1.4%)	
**M stage, n (%)**			1.00			1.00
M0	2063 (89.0%)	380 (96.2%)		794 (94.2%)	2005 (90.8%)	
MX	1 (0.0%)	0 (0.0%)		0 (0.0%)	1 (0.0%)	
Missing	253 (10.9%)	15 (3.8%)		49 (5.8%)	201 (9.1%)	
**Cancer stage, n (%)**			0.44			0.110
1	728 (31.4%)	137 (34.7%)		191 (22.7%)	434 (19.7%)	
2	785 (33.9%)	128 (32.4%)		332 (39.4%)	861 (39.0%)	
3	804 (34.7%)	130 (32.9%)		320 (38.0%)	912 (41.3%)	
**Tumor location, n (%)**			<0.001			<0.001
Colon	1574 (67.9%)	142 (35.9%)		661 (78.4%)	1577 (71.5%)	
Rectum	741 (32.0%)	253 (64.1%)		182 (21.6%)	629 (28.5%)	
Missing	2 (0.1%)	0 (0.0%)		0 (0.0%)	1 (0.0%)	
						
**Neoadjuvant therapy, n (%)**	407 (17.6%)	182 (46.1%)	<0.001	127 (15.1%)	494 (22.4%)	<0.001
Missing	16 (0.7%)	0 (0.0%)		0 (0.0%)	18 (0.8%)	
						
**Adjuvant therapy, n (%)**	176 (7.6%)	36 (9.1%)	0.350	78 (9.3%)	311 (14.1%)	<0.001
**Type of surgery, n (%)**			<0.001			0.007
Ileocecal resection	2 (0.1%)	0 (0.0%)		3 (0.4%)	7 (0.3%)	
Right hemicolectomy	954 (41.2%)	85 (21.5%)		378 (44.8%)	853 (38.6%)	
Left hemicolectomy	79 (3.4%)	11 (2.8%)		76 (9.0%)	208 (9.4%)	
Transverse colon resection	6 (0.3%)	1 (0.3%)		13 (1.5%)	43 (1.9%)	
Sigmoid colon resection	424 (18.3%)	29 (7.3%)		102 (12.1%)	270 (12.2%)	
Total Colectomy	25 (1.1%)	1 (0.3%)		49 (5.8%)	100 (4.5%)	
Hartmann's procedure	65 (2.8%)	19 (4.8%)		50 (5.9%)	122 (5.5%)	
Anterior resection	510 (22.0%)	94 (23.8%)		106 (12.6%)	365 (16.5%)	
						
Abdominoperineal excision	252 (10.9%)	155 (39.2%)		66 (7.8%)	239 (10.8%)	

ASA, American Society of Anesthesiologists.

**Table 2:** Crude outcomes. Postoperative complications include all recorded complications (Clavien-Dindo grade I-V), the subdivided complications (cardiovascular, infectious, surgical and neurological) only Clavien-Dindo grade >IIIa.

	Minimally Invasive Surgery			Open Surgery		
	No Epidural Anesthesia N = 2317	Epidural Anesthesia N = 395	*P*-value	No Epidural Anesthesia N = 843	Epidural Anesthesia N = 2207	*P*-value
Length of stay			<0.001			0.150
Median (IQR)	4.0 (3.0–7.0)	6.0 (4.0–8.5)		7.0 (5.0–10.0)	7.0 (5.0–10.0)	
Missing	14 (0.6%)	0 (0%)		0 (0%)	14 (0.6%)	
Overall postoperative complications, n (%)	406 (17.5%)	99 (25.1%)	<0.001	213 (25.3%)	622 (28.2%)	0.110
Cardiovascular complications, n (%)	28 (1.2%)	7 (1.8%)	0.500	23 (2.7%)	51 (2.3%)	0.590
Infectious complications, n (%)	118 (5.1%)	36 (9.1%)	0.002	79 (9.4%)	210 (9.5%)	0.960
Surgical complications, n (%)	74 (3.2%)	13 (3.3%)	1.00	18 (2.1%)	77 (3.5%)	0.071
Neurological complications, n (%)	4 (0.2%)	0 (0.0%)	0.910	3 (0.4%)	8 (0.4%)	1.00
Other complications, n (%)	182 (7.9%)	43 (10.9%)	0.044	90 (10.7%)	275 (12.5%)	0.175
Missing, n (%)	0 (0.0%)	0 (0.0%)		0 (0.0%)	1 (0.0%)	
30-day mortality, n (%)	16 (0.7%)	2 (0.5%)	0.930	8 (0.9%)	23 (1.0%)	0.980
90-day mortality, n (%)	24 (1.0%)	4 (1.0%)	1.00	17 (2.0%)	39 (1.8%)	0.760
3-year mortality, n (%)	234 (10.1%)	28 (7.1%)	0.075	138 (16.4%)	350 (15.9%)	0.770

## 3 Results

### 3.1 Epidural analgesia and minimally invasive surgery

Of the 11,192 patients who underwent an elective, curative operation for colorectal cancer during the study period, 5762 (51.4%) met the inclusion criteria, of whom 2712 (47.4%) underwent an MIS colorectal resection. There was no statistically significant difference in age, sex, ASA classification, or cancer stage between the EA^+^ and EA^−^ cohorts among patients who underwent MIS procedures. EA^+^ patients were more likely to have been diagnosed with rectal cancer (64.1% vs 32.0%, p < 0.001) and received neo-adjuvant therapy to a larger extent (46.1% vs 17.6%, p < 0.001) (Table 1). There was no statistically significant difference in crude 30-day, 90-day, or 3-year mortality between the cohorts; however, there was a higher prevalence of postoperative complications among EA^+^ patients (25.1% vs. 17.5%, p < 0.001) (Table 2). In MIS, EA + significantly increased the median duration of hospital stay [days (IQR) 6.0 (4.0–8.5) vs. 4.0 (3.0–7.0) days) (p < 0.001)] (Table 2). After adjusting for age, sex, ASA classification, type of surgery, neo-adjuvant therapy, tumor location, and cancer stage, epidural analgesia was not associated with a reduction in postoperative complications or postoperative mortality in patients subjected to minimally invasive surgery (Table 3).

**Table 3:** Incidence Rate Ratio and outcomes for patients who received epidural anesthesia.

	Minimally Invasive Surgery		Open Surgery	
	IRR (95% CI)	*P*-value	IRR (95% CI)	*P*-value
Postoperative complication	1.03 (0.82–1.30)	0.801	1.08 (0.92–1.26)	0.359
Cardiovascular complication	1.24 (0.45–3.44)	0.686	1.09 (0.60–1.97)	0.794
Infectious complication	1.41 (0.92–2.16)	0.111	0.98 (0.74–1.29)	0.886
Surgical complication	1.00 (0.51–1.94)	0.995	1.40 (0.78–2.51)	0.261
Neurological complication	N/A	N/A	6.22 (0.04–9.61)	0.487
30-day mortality	0.74 (0.08–6.93)	0.802	0.84 (0.33–2.13)	0.719
90-day mortality	1.04 (0.26–4.20)	0.960	0.98 (0.49–1.98)	0.964
3-year mortality	0.71 (0.48–1.07)	0.101	1.02 (0.83–1.24)	0.877

Poisson regression model with robust standard errors. The reference group for each analysis is patients who did *not* receive epidural anesthesia. Model adjusted for age, sex, ASA classification, type of surgery, neo-adjuvant therapy, tumor location, and cancer stage. Multiple imputation by chained equations was employed to compensate for missing data; logistic regression was used for binary variables, and a proportional odds model was used for ordinal variables.

IRR, Incidence rate ratio; ASA, American Society of Anesthesiologists.

### 3.2 Epidural analgesia and open surgery

Among the 3050 patients who underwent open surgery, patients with epidural analgesia were more likely to be male (53.5% vs. 49.3%, p = 0.046) and operated for rectal cancer (28.5% vs. 21.6%, p < 0.001). There were more patients with ASA class ≥3 in EA^−^ than EA^+^ (43.2% vs. 37.8% p = 0.007) and EA^−^ were less likely to have received neo-adjuvant therapy than EA^+^ (15.1% vs. 22.4%, p < 0.001) (Table 1). EA was not associated with a reduction in the incidence of 30-day, 90-day, or 3-year mortality, or in postoperative complications (Table 2). After adjustment for covariates in the Poisson regression analyses there was no difference in the risk of adverse outcomes between the cohorts (Table 3).

## 4 Discussion

In this large cohort study, no association could be detected between EA and postoperative complications or better survival (up to 3 years) in patients undergoing surgery for colorectal cancer, either by minimally invasive or open surgical approach.

Current guidelines, specifically the clinically widespread ERAS pathway guidelines, emphasize EA after open abdominal surgery, for better pain relief and to facilitate early postoperative mobilization [7]. EA is presumed to decrease postoperative complications associated with immobility, such as pneumonia and deep vein thrombosis [15], and to ameliorate gastrointestinal motility after abdominal surgery facilitating earlier oral nutrition intake [3,16]. Furthermore, EA has been shown to reduce the stress response caused by the surgical trauma [1], as well as postoperative immune suppression [17,18]. Despite these reported benefits, EA is currently only recommended after open surgery, and not after minimally invasive procedures [7].

The reasons for the controversy surrounding the anticipated benefits of EA are multifold. The risk of failure of adequate analgesia with EA is estimated to be as high as 13%–40% [19], and its failure can cause severe pain and necessitate the use of rescue analgesics. Often this takes the form of systemic opioids, along with all their well-recognized disadvantages. Patients receiving EA also have a higher incidence of pruritus and hypotension, which may cause discomfort and prevent early mobilization which could potentially prolong postoperative recovery [20]. Although rare complications, hemorrhagic and infectious complications related to neuraxial blockade, which are disastrous events for the affected patients, can occur [21]. During the last decade, multimodal analgesia as an alternative to EA has been proposed and investigated. Several studies have shown comparable postoperative pain management in MIS procedures and open abdominal surgery using the transversus abdominal plane block [[22], [23], [24]]. Recently, Ng Cheong Chung et al. have shown that a multimodal approach including intrathecal morphine, paravertebral and rectus sheath block provides comparable analgesia to thoracic EA in transthoracic oesophagectomy [25]. A meta-analysis of 29 randomized controlled trials, including 2059 patients, demonstrated that postoperative pain control after abdominal surgery is comparable to epidural analgesia when pre-peritoneal wound catheters are used. Further, patient satisfaction was higher with pre-peritoneal wound catheters than epidural analgesia [26]. Intraperitoneal administration of local anesthetics has also shown promising results in abdominal surgery [27]. Consequently, the role of EA as the gold standard for postoperative pain management after elective colorectal cancer surgery has been questioned [28].

Previous studies have yielded conflicting results regarding the effects of EA on postoperative morbidity and mortality. Turunen et al. observed better pain relief in patients receiving EA during 48 h after laparoscopic sigmoidectomy. However, there was no difference in overall complication rate or recovery [29], which was similar to our findings in the present study. In contrast, Marret et al. found that patients receiving EA for colorectal surgery experienced more pruritus, urinary retention and hypotensive episodes [30], which could increase the risk of postoperative complications and hospital length of stay. In a more recent RCT including 122 patients randomized to EA or patient-controlled opioid analgesia within an ERAS program, recovery was similar between the groups, while overall complications and the need for vasopressors were more frequent in the EA-group [31]. In the current study, we found patients with EA undergoing MIS had a prolonged length of hospital stay, but not those undergoing open surgery and receiving EA, which was similar to the results of Borzellino et al. [32] However, it is important to mention that in the current study more patients who had EA for MIS had rectal cancer, possibly affecting the outcome. Regarding mortality benefits of EA, in one meta-analysis based on a variety of surgical procedures, Popping et al. showed reduced postoperative morbidity and mortality when EA was used compared to systemic opioid analgesia (OR 0.6, 95% CI 0.39–0.93) [33], confirming results from an earlier meta-analysis by Rodgers et al. [15] To detect a survival benefit of any interventions after elective colorectal cancer surgery, a large sample size is required since 30-day postoperative mortality is between 1 and 2% [34,35]. The 30-and 90-day mortality rates seen in our study correspond well with data for all patients registered in the SCRCR, indicating that our patient selection is representative. The association between EA and long-term survival after colorectal cancer surgery is another controversial and debated topic. One study found a better overall survival but only during a limited study period [36], or in a specific subset of patients undergoing rectal and not colon cancer surgery [37]. In a retrospective analysis of data from patients who were included in a prospective randomized controlled trial conducted 1992–1994, Christopherson et al. found that patients without metastases who did not receive EA for colon cancer surgery had a higher risk of death before 1.46 years after surgery (HR 4.56, 95% CI 1.4–15.42) [36]. However, the data underlying this analysis was collected over 25 years ago, and significant improvements in surgical and anesthetic techniques as well as the perioperative care have taken place since then. In contrast, several studies were not able to demonstrate any difference in overall survival [38]. In a long-term follow-up of the MASTER trial that randomized patients to epidural analgesia or systemic opioid analgesia for major abdominal cancer surgery including a variety of procedures between 1995 and 2001, the authors could not detect any difference in median recurrence-free survival (2.6 years in EA group, 2.8 years without EA, HR 0.95, 95% CI 0.76–1.17) and median survival (EA group 3.3 years, no EA 3.7 years, HR 0.95, 95% CI 0.77–1.18) [38]. In another retrospective analysis by Day et al., no difference in overall or disease-free survival at five years was evident when comparing EA to spinal analgesia and patient-controlled opioid analgesia after laparoscopic colorectal cancer surgery between 2003 and 2010 [39]. All studies published so far are retrospective or post hoc analyses of prospective randomized trials and most included only a relatively small number of patients [8]. The current study confirms these latter studies, where no association between long-term survival and EA use after surgery was detected.

There are limitations to the current study that need to be recognized. We retrieved data from the SCRCR, a prospectively collected database, including >99% of all patients diagnosed with colon or rectal cancer in Sweden. However, the Swedish Perioperative Registry (SPOR) was started in 2013, with significantly fewer hospitals contributing to it than to the SCRCR. This led to the inability to cross-reference all patients and the exclusion of 40.5% (n = 4530) of the patients operated for elective, curative colorectal cancer during the study time period, introducing a potential source of bias. However, all patients who underwent surgery in hospitals that contribute to the SPOR are included in the analysis, mitigating the risk of inclusion bias at institution level. The datasets also lack detailed information about comorbidities, which forced us to use the ASA classification as a substitute. The ASA classification does not consider the type of comorbidity but instead, crudely focuses on the cumulative comorbidity burden. No analysis relating to the dose or type of active substance, EA failure rates, use of rescue medication, or the timeframe for perioperative EA could be performed as neither database captures these data. There was also no data available pertaining to patient reported, or other assessments of, pain control.

## 5 Conclusion

Epidural analgesia, as part of the comprehensive care provided, was not associated with a reduction in postoperative complications risk or improved 30-day, 90-day, or 3-year survival after elective, curative colorectal cancer surgery. Future prospective randomized controlled studies are required in order to provide more robust evidence into the routine use of EA in colorectal cancer surgery.

## Funding

Wiebke Falk received funding from ALF funding Region Örebro County (OLL-880951).

## Provenance and peer review

Not commissioned, externally peer-reviewed.

## Research Registration Unique Identifying Number (UIN)

Name of the registry: Project database Region Örebro County.

Unique Identifying number or registration ID: 273334.

Hyperlink to your specific registration (must be publicly accessible and will be checked): https://www.researchweb.org/is/fourol/project/273334.

## Author contribution

Wiebke Falk- study design, ethical application, request of data from registries, interpretation of results, writing of the manuscript, Anil Gupta- study design, interpretation of results, final approval of the manuscript, Maximilian Peter Forssten- data analysis, final approval of the manuscript, Hans Hjelmqvist- interpretation of results, final approval of the manuscript, Gary Bass- data analysis, final approval of the manuscript, Peter Matthiessen- study design, request of data from registries, interpretation of results, final approval of the manuscript, Shahin Mohseni- study design, data analysis and interpretation, drafting of the manuscript.

## Guarantor

Wiebke Falk.

Shahin Mohseni.

## Declaration of competing interest

The authors have no conflicts of interest to disclose.
